# The Influence of Posture on Attention

**DOI:** 10.1027/1618-3169/a000567

**Published:** 2023-02-21

**Authors:** Emilie E. Caron, Laura R. Marusich, Jonathan Z. Bakdash, Reynolds J. Ballotti, Andrew M. Tague, Jonathan S. A. Carriere, Daniel Smilek, Derek Harter, Shulan Lu, Michael G. Reynolds

**Affiliations:** ^1^ Department of Psychology, University of Waterloo, Ontario, Canada; ^2^ DEVCOM U.S. Army Research Laboratory, Austin, TX, USA; ^3^ Department of Psychology and Special Education, Texas A&M University-Commerce, Commerce, TX, USA; ^4^ Department of Psychology, Bishop’s University, Sherbrook, Quebec, Canada; ^5^ Department of Computer Science and Information Systems, Texas A&M University-Commerce, Commerce, TX, USA; ^6^ Department of Psychology, Trent University, Peterborough, Ontario, Canada

**Keywords:** cognitive control, embodiment, posture, attention, standing

## Abstract

**Abstract.** Smith et al. (2019) found standing resulted in better performance than sitting in three different cognitive control paradigms: a Stroop task, a task-switching, and a visual search paradigm. Here, we conducted close replications of the authors’ three experiments using larger sample sizes than the original work. Our sample sizes had essentially perfect power to detect the key postural effects reported by Smith et al. The results from our experiments revealed that, in contrast to Smith et al., the postural interactions were quite limited in magnitude in addition to being only a fraction of the size of the original effects. Moreover, our results from Experiment 1 are consistent with two recent replications (Caron et al., 2020; Straub et al., 2022), which reported no meaningful influences of posture on the Stroop effect. In all, the current research provides further converging evidence that postural influences on cognition do not appear to be as robust, as was initially reported in prior work.


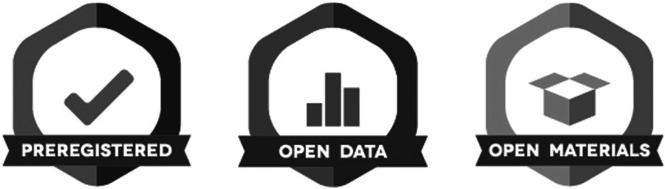


Since the introduction of *standing desks*, otherwise known as active workstations (MacEwen et al., 2015; Torbeyns et al., 2014), there has been ongoing debate regarding whether standing can positively influence an individual’s cognitive performance relative to sitting (Caron et al., 2020; Rosenbaum et al., 2017/2018; Smith et al., 2019; Straub et al., 2022). Recent work on this topic finds its roots in the theoretical framework of embodied cognition, which maintains that cognitive processes are best understood in terms of contextualized interactions among the body, brain, and environment (Matheson & Barsalou, 2018). Also at stake are practical issues regarding which postures might be optimal for information processing in various contexts such as educational and workplace environments (Harmon-Jones et al., 2011; Koepp et al., 2012; Price & Harmon-Jones, 2010).

Building on earlier research by Ebara et al. (2008) and others (e.g., Bantoft et al., 2016; Ohlinger et al., 2011; Russell et al., 2016), Rosenbaum et al. (2017/2018) reignited interest in this topic through their multi‐experiment demonstration that showed how simply standing rather than sitting can reduce interference from irrelevant stimulus dimensions in the Stroop task. The Stroop task (1935) is a cognitive task commonly used in the laboratory to capture individuals’ levels of selective attention and attentional control. During the Stroop task, participants are instructed to ignore the word and focus solely on the color of the word stimulus. That is, the task requires participants to indicate by verbal or manual response the color of a word stimulus when the color is either congruent (e.g., the word “RED” printed in the color red) or incongruent (e.g., the word “RED” printed in the color blue) with the word itself. As has been consistently observed since Stroop conducted his first experiment in 1935, individuals are slower and less accurate at identifying the color of a word stimulus on incongruent trials than on congruent and neutral ones (MacLeod, 1991; Stroop, 1935). This difference in performance between the congruent and incongruent trials – known as the *Stroop effect –* is thought to indicate a failure of selective attention in which attention is captured by the to-be-ignored word.

In their study, Rosenbaum et al. (2017/2018) had participants complete the Stroop task while both sitting and standing; they found that relative to sitting, standing was associated with a Stroop effect of a smaller magnitude. Through their interpretation of these results, the authors theorized that standing improves selective attention on the Stroop task, because standing increases “load” leading to fewer resources being available to process the distracting word. These findings led to a series of conceptual and direct replications with mixed results (e.g., Caron et al., 2020; Smith et al., 2019; and Straub et al., 2022). One group of researchers reported a successful replication demonstrating a large significant effect (Experiment 1 in Smith et al., 2019), while a series of multiple experiments conducted by others failed to find any meaningful effects (Experiments 1–5 in Caron et al., 2020; Experiment 1 in Straub et al., 2022).

Additionally, when Straub et al. (2022) meta‐analyzed effect sizes for 10 posture and Stroop effect experiments (five experiments from Caron et al., 2020; two experiments from Rosenbaum et al., 2017/2018; one experiment from Smith et al., 2019; two experiments from Straub et al., 2022), they found a small overall effect size with a confidence interval crossing below the null (Cohen’s d = 0.06, 95% CI [−0.04, 0.16]). The combination of these results raises questions about the relationship between posture and Stroop performance.

While the effect of posture on Stroop performance does not appear to be as robust as was originally reported, there remains the possibility that posture might influence other cognitive processes indexed by alternate laboratory tasks. In fact, Smith et al. (2019) found evidence of an influence of posture on performance in two other common cognitive control paradigms: task-switching (Experiment 2) and visual search (Experiment 3). Specifically, in Experiment 2 of Smith et al., participants engaged in a task-switching paradigm in which they switched between responding to the color of an upcoming stimulus and responding to its shape depending on the cue that appeared before the stimulus on a given trial. Participants completed switch trials (i.e., trials that required participants to respond to a different dimension [color/shape] than they had on the previous one) and no-switch trials (i.e., trials that required participants to respond to the same dimension as the previous one) in both the sitting and standing positions. Accuracy across these trials was subsequently compared, and the results showed a reduction in switch costs when participants were standing in comparison to when they were sitting. In Experiment 3 of Smith et al., participants completed a visual search paradigm in both the sitting and standing positions and were required to search through a set of either 4- or 8-letter stimuli to identify the target letter (S or H) from the distractor letters (U or E). In this paradigm, participants showed steeper search slopes when they were standing relative to when they were sitting.

The findings reported by Smith et al. have considerable theoretical import. Smith et al. proposed that compared to sitting, standing might increase arousal to moderate levels, which in turn increases cognitive resources that lead to better attentional selection and cognitive control. Alternatively, Smith et al. noted that standing may conjure up “mental states” typically associated with an erect posture, such as the fight-or-flight response, which might place people into a mental set that involves heightened attentional processing. Either way, the findings reported by Smith et al. suggest a wider influence of posture on cognition than was demonstrated by Rosenbaum et al., thus indicating that posture, and potentially other bodily states, might impact cognitive processes involved in various attention tasks including attentional switching and visual search.

As noted by Straub et al. (2022), if effects of posture on cognition were consistently meaningful, then there are a variety of potential applications in clinical and educational areas of work. For instance, developing and implementing a sit/stand routine might help individuals of all ages improve their behavior (Katz et al., 2015) and redirect their focus from their internal intrusive thoughts to the tasks they must complete (Benden et al., 2011; Blake et al., 2012; Koepp et al., 2012). Additionally, if postural strategies narrow an individual’s attention toward a more specific element of information (e.g., Harmon-Jones et al., 2011; Harmon-Jones & Peterson, 2009; Price & Harmon-Jones, 2010), then this might assist the acquisition of information for students presenting with attention deficit hyperactivity disorder, or perhaps individuals required to switch tasks and maintain heightened levels of attention during critical moments throughout their day (e.g., air traffic controllers or soldiers on duty).

## Present Studies

Given the theoretical and broad potential implications of the studies reported by Smith et al. (2019), here we report the results of close replications of all three of their experiments to determine how extensively posture influences cognitive processes. In Experiment 1, we examined the influence of posture (i.e., sitting vs. standing) on performance (e.g., Stroop effect) in a close replication of Smith et al.’s Stroop task. In Experiments 2 and 3, we explored the effect of posture on switch-costs in a close replication of the authors’ task-switching paradigm and on search-rates in a close replication of their visual search paradigm.

The straightforward prediction for these experiments is that we will replicate the findings reported in Smith et al. (2019). Specifically, we will obtain the standard cognitive effects typically observed in each task (i.e., a main effect of congruency in Experiment 1, main effects of switching and congruency in Experiment 2, and a main effect of set size in Experiment 3), in addition to observing that these cognitive effects interact with posture where such interactions were reported by Smith et al. Given the abovementioned failures to replicate, however, another possibility is that we will fail to obtain some, if not all, of the postural interactions reported by Smith et al.

## General Methods

Experiments 1 and 3 were conducted independently at Trent University in collaboration with the University of Waterloo and Bishop’s University, while Experiment 2 was conducted by a group of researchers at Texas A&M University - Commerce in collaboration with the U.S. Army Research Laboratory. After data collection was completed for Experiment 1, the two groups of researchers became aware of each other’s mutual interest in replicating Smith et al.'s (2019) experiments. Both groups of researchers thus decided to conduct an international multi-university and pre-registered replication of Smith et al.’s experiments to better understand the true nature of the effect of posture on cognition. As a result, the reader will notice slight differences across the study protocols (e.g., whether demographic information was collected or not).

Across all three replication experiments, we chose sample sizes that would ensure high statistical power to detect not only effect sizes of the magnitude originally reported by Smith et al. (2019) but also to detect minimum effect sizes of interest (Lakens, 2022). Table 1 summarizes the originally reported effect sizes of the Smith et al. experimental paradigms and the minimum effect sizes of interest (in addition to their corresponding statistical power) for the current replication work. Statistical power was calculated for repeated trials (Goulet & Cousineau, 2019) using SuperPower (Lakens & Caldwell, 2021; Supplemental Materials on https://osf.io/kwrjd). The minimum effect sizes of interest in the replication experiments fall within the upper end of the small range. This is a fraction of the effect sizes originally reported in Smith et al., which were in the medium-to-large range. Consequently, the minimum effect sizes of interest provide lower boundaries for estimating statistical power.

**Table 1:** Summarized results for the two-way interaction from the three Smith et al. (2019) experiments and computed statistical power (based on an *N* = 50) for the three replication experiments reported here

Experiment	Interaction of interest	Variable of interest	Smith et al.: Sample size (*N*)	Smith et al.: reported effect size (η_*p*_^2^)	Current replication: minimum effect size of interest (η_*p*_^2^)	Current replication: pre-registered sample size	Current replication: computed statistical power to obtain Smith et al. reported effect sizes (%)	Current replication: computed statistical power to obtain minimum effect size of interest (%)
Experiment 1: Stroop	Posture × Congruency	Reaction time	14	0.27	0.05	50	>99.99	>99.99
Experiment 2: Task-switching	Posture × Condition (switch vs. no-switch)	Percentage error	30	0.16	0.05	50	>99.99	99.2
Experiment 3: Visual search	Posture × Set Size	Reaction time	12	0.35	0.05	50	>99.99	99.5

Data for all experiments were analyzed using the *R* statistical software program (R Core Team, 2022). In the three experiments, posture and the experimental manipulations of cognitive control were always within-participant factors. We conducted separate repeated-measures analysis of variance for response time (RT) and percentage error (PE) data using the *EZ* package (Lawrence, 2016). In each experiment, we calculated the Bayes factor (BF) using the anovaBF function from the *BayesFactor* package (Morey et al., 2021). The BF was used to compare models with and without the previously described postural interactions of interest.

It is also important to note that Smith et al.’s (2019) data analysis took the somewhat unconventional approach of treating trials that exceeded their response deadline (1,500 ms) as *errors* rather than excluding such trials or analyzing them separately as *omissions*. We contend this approach can be problematic because it makes the assumption that the probability of making an incorrect response after the arbitrary response deadline is 100%. Two consequences of this approach are that it makes interpretation of the error data more difficult (i.e., lack of a response is treated the same as an incorrect response) and it affects other critical indicators of cognitive performance, such as those related to speed accuracy trade-offs. Despite recognizing the methodological and interpretive issues that arise from analyzing the data in this way, however, we decided to use this approach for our primary analyses, so as to ensure the closest possible replication of Smith et al. (2019). In addition, we report a conventional analysis of errors in the Appendix, as well as note in the results any instances where the two approaches differ substantially.

## Experiment 1

In Smith et al. (2019) Experiment 1, participants completed a modified version of the Stroop task in both the sitting and standing conditions. In the authors’ version of the task, participants responded to the color of two color word stimuli (“RED” and “GREEN”) on congruent and incongruent trials. They also responded to the color of the stimuli on neutral (i.e., “XXX” or “XXXX”) trials. Smith et al. noted that the Stroop effect had been eliminated when participants completed the task in the standing condition compared to when they completed the task in the sitting condition. To foreshadow, we failed to replicate Smith et al.’s posture by congruency interaction. Below, we provide a detailed description of our procedural design and findings.

### Method

#### Participants

We recruited 50 Trent University undergraduate students for Experiment 1. Our rationale was to collect a sample size that aligned with the sample collected by Rosenbaum et al. (2017/2018) in their Experiment 3 and Caron et al. (2020) in their Experiment 5. Table 1 provides detailed information regarding the estimate of the effect size for Experiment 1. All participants met the inclusion criteria (see the Results section for details) and reported normal to corrected-to-normal vision as well as normal color vision. Finally, participants received partial course credit toward an eligible class.

#### Stimuli and Apparatus

The stimuli consisted of the color words “RED” or “GREEN” presented in uppercase and in a color that was either congruent (e.g., “RED” printed in red) or incongruent (e.g., “RED” printed in green) with the word itself. The experiment also consisted of neutral stimuli that contained a string of either 3 or 5 X’s matching the number of characters in each color word (e.g., “XXX” for the color word “RED” or “XXXXX” for the color word “GREEN”). The string length of the neutral stimuli was counterbalanced across colors so that strings of both lengths were presented an equal number of times in both red and green colors. The stimuli subtended approximately 0.6° high × 1.8° to 2.8° wide in visual angle and were presented on a black (RGB: 0, 0, 0) background in Courier New and 24-point font. Preceding each color‒word stimulus, a white fixation cross was presented in the center of the display for 500 ms. The cross would then be replaced by a color‒word stimulus. Participants were instructed to respond, via button press, to the color of the stimuli as quickly and accurately as possible while ignoring the meaning of the color word itself. If participants pressed the wrong key on the keyboard or did not respond within 1,500 ms, an error tone was made. A 1,500-ms inter‐trial‐interval separated each trial.

The experiment consisted of four practice blocks in addition to eight experimental ones, amounting to a total of 12 blocks. Each block, including the practice ones, contained 12 congruent, 12 neutral, and 12 incongruent trials. Participants completed six blocks in the sitting condition (i.e., two practice and four experimental blocks) and six in the standing condition (i.e., two practice and four experimental blocks). In all, there were 144 experimental trials per postural condition. The assignment of postural condition was counterbalanced across participants. The order of block presentation was random, and participants were allowed a brief break between each block. The experiment was programmed in E-Prime: https://pstnet.com/products/e-prime/.

A DELL XPS 8930 computer with Windows 10 Pro and an NVIDIA Geforce GTX 1050TI video card and a DELL 24-inch Gaming Monitor (Model S2421HGF) with a native resolution of 1920 × 1,080 (running at 120 Hz) were used to conduct the experiment. In both the sitting and standing conditions, the stimuli were presented at eye level, and data were collected using a keyboard that participants held vertically to their chest. The computer and monitor were set on an Ergotron^®^ WorkFit™-TX standing desk converter that was placed on an Ikea Jerker desk. In the standing condition, participants stood with their feet hip-width apart, whereas in the sitting condition, they sat on an ergonomic office chair. Participants’ viewing distance was held constant at approximately 76.2 cm by an adjustable chinrest.

### Results

**Figure 1:**
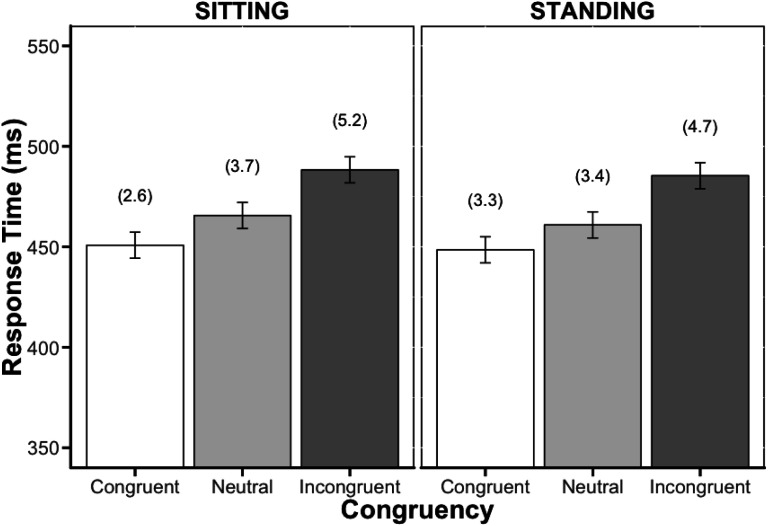
Mean response times and percentage error (presented in parentheses) as a function of posture and congruency from Experiment 1. Error bars represent the 95% confidence intervals calculated according to Loftus and Masson (1994).

Figure 1 presents the mean response time (RT) and percentage error (PE) data, which were averaged across the 50 analyzed participants and the two posture conditions. As a pre-registered inclusion criterion, participants were excluded from the analyses if they lost more than 20% of the data prior to outlier trimming. All participants maintained 80% or more of their data, and thus, no participant was removed as a result of this criterion. Of the participants’ overall data, 0.01% were removed due to premature responding (i.e., RTs faster than or equal to 100 ms). To ensure that only correct responses were included within the RT data analysis, 3.79% of the remaining trials were removed due to errors such as the trials timing out (i.e., participants failed to make a response within 1,500 ms of the stimulus presentation) or trials on which participants named the word of the stimuli as opposed to its color. Finally, a recursive data trimming procedure was subsequently employed on the remaining correct RT trials and resulted in the removal of an additional 2.11% trials. This data trimming procedure consisted of calculating a unique outlier criterion for each participant in each cell based on the number of observations per cell (see Van Selst & Jolicoeur, 1994, in Caron et al., 2020).

#### Response Time

In all, there was a significant main effect of congruency, *F*(2, 98) = 22.47, *MSE* = 1,578.33, *p* < .001, η_*p*_^2^ = .314. However, no significant main effect of posture, *F*(1, 49) = 0.26, *MSE* = 3,188.11, *p* = .614, η_*p*_^2^ = .005, nor interaction of posture by congruency, *F*(2, 98) = 0.08, *MSE* = 520.96, *p* = .922, η_*p*_^2^ < .002, BF = 15.63, *p*BIC(H0|D) = 0.06, was observed. BF results show that there was 16 times more evidence for the null (no observable posture by congruency interaction in the model) than for the alternative hypothesis (observable interaction included in the model). Following Smith et al. (2019), the Stroop effect (incongruent–congruent) was examined separately for the sitting and standing conditions. The 38-ms Stroop effect in the sitting condition was significant, *t*(49) = 5.12, *p* < .001, as was the 37-ms Stroop effect in the standing condition, *t*(49) = 4.38, *p* < .001.

#### Percentage Error

Regarding the PE data, we continued to observe a significant main effect of congruency, *F*(2, 98) = 11.60, *MSE* = 9.22, *p* < .001, η_*p*_^2^ = .191. However, similar to the RT data, we found no significant main effect of posture, *F*(1, 49) = 0.007, *MSE* = 16.56, *p* = .934, η_*p*_^2^< .001, indicating that the 1.4% Stroop effect in the standing condition did not significantly differ from the 2.6% Stroop effect in the sitting condition. Nor did we find a significant interaction of posture by congruency, *F*(2, 98) = 1.59, *MSE* = 6.23, *p* = .209, η_*p*_^2^ = .031, BF = 6.58, *p*BIC(H0|D) = 0.15. The results favored the null hypothesis (model with no interaction) approximately 7 times more than the alternative (model with the interaction).

### Discussion

In a sample of 50 participants, we obtained the standard Stroop effect, wherein participants were slower and less accurate when responding to the incongruent trials than they were when responding to the congruent and neutral trials. Critically, it did not matter whether participants completed the Stroop task while sitting or standing. Participants’ performance did not significantly differ based on posture (sitting vs. standing), nor did posture significantly influence the magnitude of the Stroop effect.

## Experiment 2

Here, we conducted a replication of Smith et al.’s (2019) Experiment 2 (task-switching paradigm). Participants were required to respond to the color of the stimuli, or its shape, depending on the preceding cue. Similar to above, participants completed a portion of the task while sitting down and another while standing up. The original findings of Smith et al. revealed that switch costs (i.e., the observable reduction in accuracy when participants responded to a trial that mismatched the preceding one compared to when they responded to a trial that matched the preceding one) were reduced when participants were standing relative to when they were sitting. Again, we were unsuccessful at replicating the postural interaction observed by Smith et al. in their Experiment 2. Note, the main postural interaction of interest in Experiment 2 is for percentage error, whereas the main postural interaction of interest in Experiments 1 and 3 is for RT.

### Method

#### Participants

Fifty-seven Texas A&M University–Commerce undergraduate students were recruited for Experiment 2. Six participants were removed from the analysis because they did not meet the inclusion criterion (see the Results section for details). The mean age of participants was 20.22 years (*SD* = 1.74). This sample size coincides with the sample size from Experiment 1 in this paper as well as with the sample size from Rosenbaum et al.’s (2017/2018) Experiment 3, and Caron et al.’s (2020) Experiment 5. Refer to Table 1 for the estimated effect size for Experiment 2. Participants reported normal to correct-to-normal vision and normal color vision and received partial course credit toward an eligible class.

#### Stimuli and Apparatus

The experiment consisted of a series of trials, each of which began with a cue (i.e., a 25° × 25° square) that was displayed in the center of a black screen (RGB: 0, 0, 0) for 1,000 ms. Depending on the cue counterbalance, participants had to either identify the color or shape of the stimulus that followed. In half of the counterbalance sequences, when the border of the square (cue) was a solid white line (RGB: 255, 255, 255), participants were required to identify the color of the upcoming stimulus. When the border of the square was a dashed line, however, participants had to identify the stimulus’ shape. The cues and the corresponding responses were reversed in the rest of the counterbalance sequences. The appearance sequence of the cue was counterbalanced across participants. The target stimulus appeared 1,000 ms after the presentation of the cue in the center of the screen. One of two target stimuli (a triangle or a square) would appear in either yellow (RGB: 255, 255, 0) or blue (RGB: 26, 88, 255) and subtended about 12° × 12° in visual angle.

Participants were required to complete nine blocks (one practice block and eight experimental ones), and after each block, participants were given a short break. Four blocks were completed while sitting and four while standing, and the assignment of posture was counterbalanced across participants. The practice block, which participants completed in the first position they were assigned to, consisted of 12 untimed and 24 speeded trials, while the experimental blocks each consisted of one buffer trial followed by 48 test trials. In all, there were 192 experimental trials per postural condition. No-switch trials occurred when the current trial contained the same task as the one that occurred before it, whereas switch trials occurred when the task on the current trial did not match the task on the trial that occurred before it. Both the target stimuli and the order of the switch and no-switch trials were randomly assigned; however, we ensured there were an equal number of switch and no-switch trials. Participants responded via button press, as quickly and accurately as possible, to either the color of the target stimuli or its shape depending on the cue. Additionally, each button was assigned either a color or a shape. As a result, on all trials, the stimulus presented could be congruent or incongruent with the button press. That is to say, the stimulus color and shape could have the same button response (e.g., congruent) or a different button response (e.g., incongruent). If participants pressed the wrong button or did not press a button within 1,500 ms, they heard an error tone and saw an error message that remained on the screen for 5,000 ms. The next trial appeared 200 ms following a correct response or an error feedback message. This experiment was programmed in PsychoPy (Peirce et al., 2019).

A desktop PC and a DELL monitor with a display resolution of 1,680 × 1,050 and at 60 Hz were used to conduct this experiment. Both the computer and monitor sat on an adjustable desk (Electric Quick-Install Height Adjustable Desk EC9), which was adjusted to be positioned so that the stimuli were presented at eye-level for each participant in both conditions. In the sitting condition, participants sat in a wheel-less, hard-backed chair. Data were collected using MK-SV-1 LabHackers MilliKey SV-1 USB Response Box. Participants held one button response box in each of their hands. They were told not to put their hands on the desk, not to lean on the desk, not to lean on one leg, or sway their body from side to side. After 16 counterbalance sequences, the response box originally held in participants’ left hand was switched to the right hand and vice versa (see the Instructions Folder). Participants’ viewing distance was fixed, using a chinrest, at 57 cm. Participants’ neck, torso, arms, and shoulder postural positions were also tracked by Kinect for exploratory purposes but were not analyzed in this study. As the desk blocked the leg joint positions, we do not have leg joints data.

### Results

**Figure 2:**
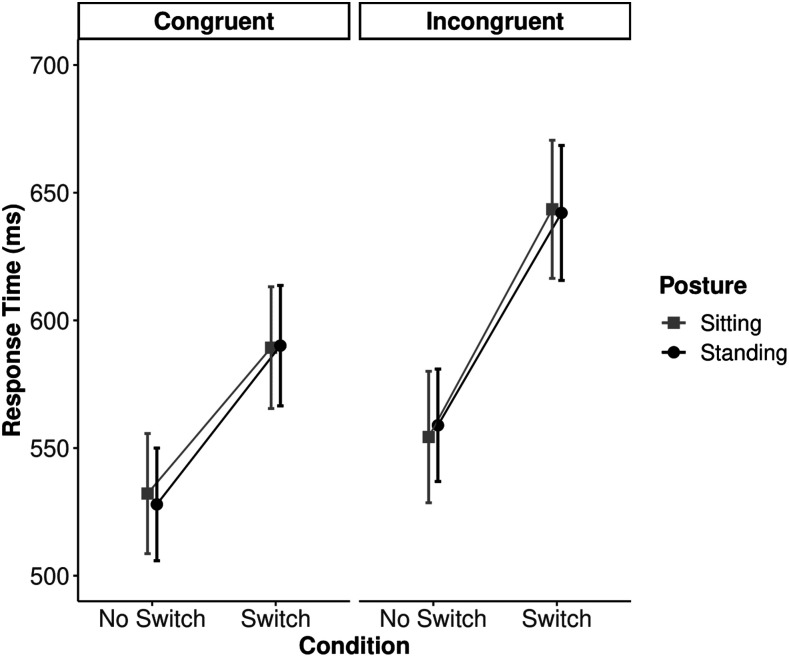
Mean response times by congruency, condition, and posture. Error bars represent the 95% confidence intervals calculated according to Cousineau et al. (2021).

**Figure 3:**
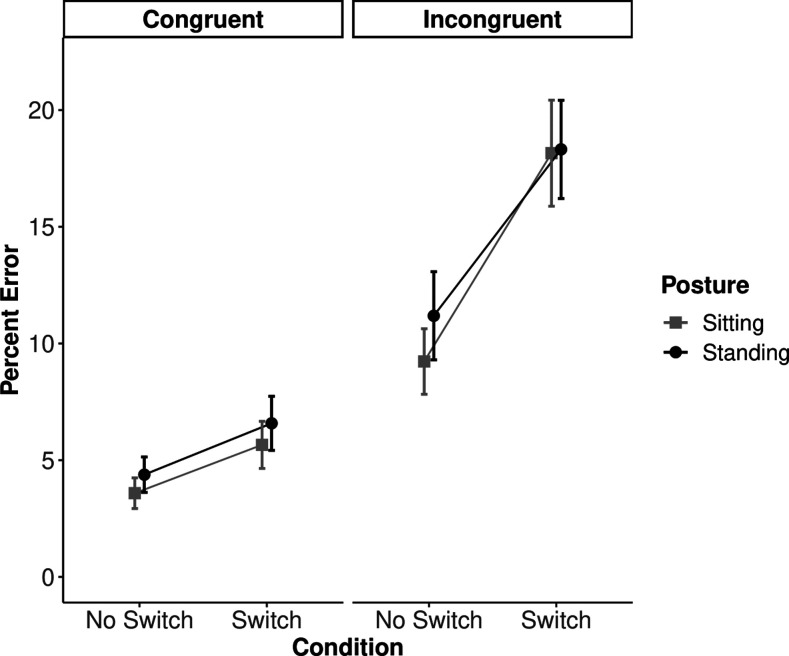
Mean percentage error by congruency, condition, and posture. Error bars represent the 95% confidence intervals calculated according to Cousineau et al. (2021).

Mean RT and PE data are shown in Figures 2 and 3. Prior to conducting the analyses, data from six participants were removed for not meeting our pre-registered inclusion criteria (i.e., their accuracy fell below 80%). This resulted in a final sample size of *N* = 51. Only a single trial (0.005% of the total data) was removed due to premature responding (RT faster than 100 ms).

Furthermore, and similar to Experiment 1, trials that timed out (i.e., responses made after 1,500 ms of the stimulus presentation) or trials in which participants incorrectly identified the target were considered errors and thus excluded from the RT data analyses. This resulted in the removal of 9.63% of the remaining trials. As in Experiment 1, the remaining RT trials were submitted to a modified recursive data trimming procedure (Van Selst & Jolicoeur, 1994), which varies the outlier criterion for each participant per cell based on the number of observations in the respective cells (see Van Selst & Jolicoeur, 1994 in Caron et al., 2020). This resulted in the removal of 2.14% of the remaining correct RT data.

#### Response Time

The analysis of RT revealed a significant main effect of switching, *F*(1, 50) = 130.17, *MSE* = 4,166.22, *p* < .001, η_*p*_^2^ = .72, indicating that it took participants significantly less time to respond to the no-switch trials than to switch trials. Figure 2 also demonstrated a significant main effect of congruency, *F*(1, 50) = 48.98, *MSE* = 3,302.21, *p* < .001, η_*p*_^2^ = .50; however, there was no main effect of posture, *F*(1, 50) < .0001, *MSE* = 9,922.05, *p* = .10, η_*p*_^2^ < .001. The analysis of RT also revealed a significant congruency by switching interaction, *F*(1, 50) = 14.32, *MSE* = 1,252.33, *p* < .001, η_*p*_^2^ = .223; however, we failed to find a significant switching by posture interaction on RT, *F*(1, 50) < .001, *MSE* = 1,519.08, *p* = .95, η_*p*_^2^ < .001, BF = 7.10, *p*BIC(H0|D) = 0.14. The results favored the null hypothesis (model with no interaction) approximately 7 times more than the alternative (model with the interaction). There were no other significant interactions.

#### Percentage Error

The results from the PE data revealed a significant main effect of switching, *F*(1, 50) = 92.04, *MSE* = 28.61, *p* < .001, η_*p*_^2^ = .648, indicating that participants were significantly more accurate on no-switch trials than they were on switch trials (Figure 3). Figure 3 also showed a significant main effect of congruency, *F*(1, 50) = 99.66, *MSE* = 86.07, *p* < .001, η_*p*_^2^ = .67; however, no main effect of posture was observed, *F*(1, 50) = 1.06, *MSE* = 88.41, *p* = .31, η_*p*_^2^ = .02. Importantly, while the results of the congruency by switching interaction were consistent with Smith et al. (2019), *F*(1, 50) = 58.43, *MSE* = 15.15, *p* < .001, η_*p*_^2^ = .54, the current study failed to replicate Smith et al.’s key finding of a switching by posture interaction, *F*(1, 50) = .74, *MSE* = 24.00, *p* = .40, η_*p*_^2^ = .015, BF = 6.30, *p*BIC(H0|D) = 0.16. The results favored the null hypothesis (model with no interaction) approximately 6 times more than the alternative (model with the interaction). No other significant interactions were observed. Analyses using a conventional method of classifying errors did not meaningfully differ from these results (see the Appendix section).

### Discussion

The current experiment failed to replicate the effects of posture on task-switching as reported in Smith et al. (2019). In Experiment 2, only the main effect of switching and the main effect of congruency were found. Participants were both faster and more accurate when responding to the no-switch trials than to the switch trials, and when responding to the congruent trials compared to the incongruent ones. While we did find significant interactions between switching and congruency for both RT and PE, we did not find any meaningful effects of standing on task-switching.

## Experiment 3

In their Experiment 3, Smith et al. (2019) had participants complete a visual search task while sitting and standing. The task consisted of trials with displays containing one of two set sizes (i.e., four or eight stimuli). Across both their RT and PE data analyses, the authors found a difference in search slopes in the standing condition compared to the sitting condition. Here, we conducted a replication of Smith et al.’s Experiment 3. While we found an effect of posture on set size in the PE data, this effect seems rather spurious and less robust than what Smith et al. initially reported. Moreover, we failed to find an effect of posture on set size in the RT analyses.

### Method

#### Participants

In line with Experiments 1 and 2, we recruited 50 Trent University undergraduate students for Experiment 3. All participants met the inclusion criteria (see the Results section for details) and reported normal to corrected-to-normal vision as well as normal color vision and received partial course credit toward an eligible class. Refer to Table 1 for information regarding the estimated effect size for Experiment 3.

#### Stimuli and Apparatus

Each trial began with a fixation cross (+) appearing at the center of the screen for 1,000 ms and subtending 1.13° × 1.13° visual angle. Subsequently, a search display appeared, containing a set of either four or eight block letter stimuli presented in black font (RGB: 0, 0, 0) on a white screen (RGB: 255, 255, 255). The letters subtended approximately 2.26° high × 1.13° wide, and within each search display, participants were presented one of the two target letters (H or S), among a series of distractor letters (either E or U). The presentation of the target and distractor letters, as well as the *Set Size* (i.e., either four or eight letter stimuli on the screen) were factorially combined and selected randomly without replacement so that each combination of factors occurred equally often. Furthermore, for each trial, the target and distractor letters were randomly assigned a location on a 5 × 5 grid centered on the screen. All letter stimuli were distanced at least 0.57° apart. Finally, on either side of fixation, the grid extended 10° visual angle horizontally and 8° visual angle vertically. Participants were instructed to identify, via button press, the target as quickly and accurately as possible. The search display remained on the screen until a response was recorded, after which an inter-trial-interval of 2,000 ms occurred. If participants responded too quickly (i.e., within 100 ms) on the trial, a visual feedback screen with the words “Too fast!” appeared. If they were too slow to respond (i.e., did not respond within 1,500 ms), they received the visual feedback “Too slow!,” and if participants pressed the wrong key, the feedback that appeared mentioned “Wrong key pressed!”

The experiment consisted of one practice block^1^ in addition to four experimental ones, amounting to five blocks altogether. The practice block contained eight trials, while the experimental block contained 64 trials, with an equal number of trials of each set size per block. Every participant completed the practice block while sitting. Regarding the experimental trials, participants completed two blocks in the sitting condition and two in the standing condition, and the order of postural condition was counterbalanced across participants. In all, there were 128 experimental trials per postural condition, and the order of block presentation was random. At the end of each block, participants were allotted a brief break before the next block. The experiment was programmed in E-Prime.

The procedure and apparatus used in this experiment are identical to the ones used in Experiment 1 with the exception of the following. Data were collected using 3-cm diameter USB buttons (usbbutton.com), and participants’ viewing distance was held constant at 90 cm with their chin resting on a chinrest. Additionally, as in Experiment 2, participants were informed that they could have their arms in a relaxed fashion without placing their hands on the desk.

### Results

**Figure 4:**
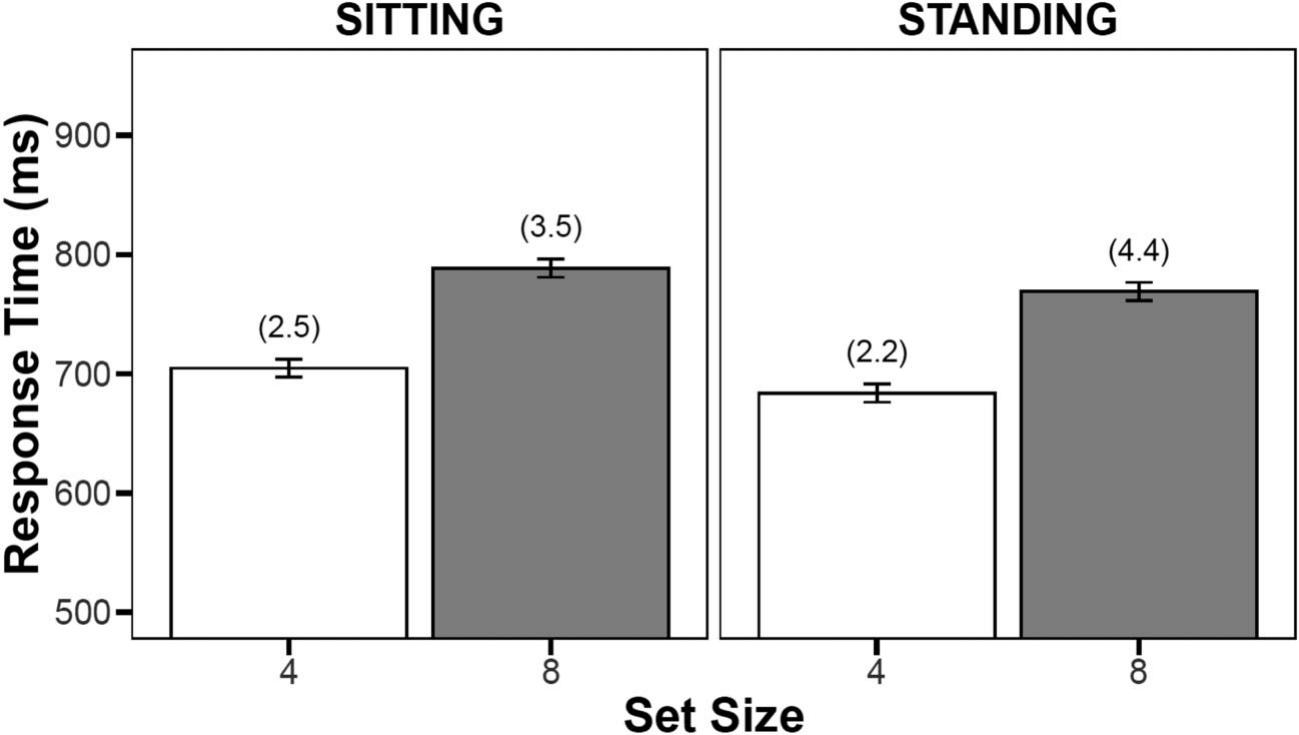
Mean response times and percentage error (presented in parentheses) as a function of Posture and Congruency from Experiment 3. Error bars represent the 95% confidence intervals calculated according to Loftus and Masson (1994).

Mean RT and PE data are presented in Figure 4. Following the pre-registered criteria, prior to data trimming, all participants maintained a minimum of 80% of their data, and thus, no participant was removed for having lost more than 20% of the data due to one or a combination of missing data, errors, and/or spoiled trials. Additionally, no trial responses were removed for being excessively short (RTs <100 ms). Prior to analyzing the RT data, trials where an error was made (i.e., trials in which participants failed to make a response within 1,500 ms of the stimulus presentation or incorrectly identified the target; 3.15%) were also excluded. Of the remaining correct RT trials, 1.34% were removed after having been submitted to the Van Selst and Jolicoeur (1994) recursive data trimming procedure employed in Experiments 1 and 2. This method ensures that the outlier criterion is individually obtained for every participant in every cell based on the total observations per cell (see Van Selst & Jolicoeur, 1994, in Caron et al., 2020).

#### Response Time

In all, there were significant main effects of posture, *F*(1, 49) = 6.54, *MSE* = 3,137.52, *p* = .014, η_*p*_^2^ = .118, and set size, *F*(1, 49) = 373.76, *MSE* = 956.39, *p* < .001, η_*p*_^2^ = .884. However, the interaction of posture by set size was not statistically significant, *F*(1, 49) = 0.03, *MSE* = 727.64, *p* = .861, η_*p*_^2^ < .001, BF = 5.11, *p*BIC(H0|D) = 0.20. The BF results favored the null hypothesis approximately 5 times more than the alternative. The search rate while standing was 21.31 ms/item [*t*(49) = 16.69, *p* < .001], and the search rate while sitting was 20.97 ms/item [*t*(49) = 13.06, *p* < .001].

#### Percentage Error

The results from the PE analyses reveal a significant effect of set size, *F*(1, 49) = 18.47, *MSE* = 7.01, *p* < .001, η_*p*_^2^ = .274, but no significant main effect of posture, *F*(1, 49) = 0.76, *MSE* = 4.65, *p* = .388, η_*p*_^2^ = .015. Regarding the significant posture by set size interaction, *F*(1, 49) = 4.61, *MSE* = 4.45, *p* = .037, η_*p*_^2^ = .086, BF = 0.94, *p*BIC(H0|D) = 1.06, these initial analyses of the results indicate that the 1.00% PE difference across set size in the standing condition was significantly different from the 2.20% PE difference across set size in the sitting condition. Notably, however, the BF results showed near equivalence for the alternative (model with the interaction) and null hypothesis (model with no interaction), with the alternative favored by only 0.94 times the null.

Additional examination of these results revealed that the statistically significant posture by set size interaction effect is entirely a consequence of treating responses that exceed the response criterion (1,500 ms) as errors (see the Appendix for details). When only traditional errors (trials on which participants report the wrong target as present in the display) are included in the analysis, the interaction does not reach statistical significance (additional analyses are accessible at https://osf.io/kwrjd). Moreover, the interaction is absent when the response criterion is increased from 1,500 to 1,750 ms or 2,000 ms, and trials are excluded based on this new criterion. None of these alternative approaches resulted in meaningful changes to the RT analyses. This suggests that the interaction appears to be a consequence of the combination of the choice to code long RTs as errors and the arbitrary definition of a long RT as 1,500 ms.

### Discussion

Experiment 3 revealed that participants took longer to find the target when the display contained a set size of eight than when it contained a set size of four. However, participants were not slower to search through the display (regardless of the displays’ set size) when they were standing in comparison to when they were sitting. Finally, we failed to find an influence of posture (sitting vs. standing) on difference scores in RT across set sizes (i.e., search slopes).

These findings suggest that relative to sitting, standing did not lead to a reduction in participants’ RT search slope. We do note however, a significant influence of posture (sitting vs. standing) on PE search slopes. That is, after analyzing data using Smith et al.’s (2019) approach to classifying errors, we find that participants displayed a greater increase in PE across increases in set size when they were standing compared to when they were sitting. Importantly, after analyzing the data using an approach to classifying errors typically employed for these types of tasks (i.e., the approach that does not include non‐responses in the computation of the error rate), we fail to find a significant influence of posture (sitting vs. standing) on difference scores in RT and PE, respectively (see the Appendix section).

## General Discussion

The primary goal of this paper was to replicate the effect of posture (sit vs. stand) on cognitive performance obtained by Smith et al. (2019) across their three attention paradigms: a Stroop task, a task-switching paradigm, and a visual search task. Upon closely replicating these three experiments, we consistently failed to observe the key findings of interest. That is, we were unable to obtain meaningful effects of posture on the magnitude of the Stroop effect in Experiment 1, on switch costs in Experiment 2, or on the search rate in Experiment 3. The only instance in which we observed a small influence of posture on performance is in Experiment 3 when we analyzed the percentage error data using Smith et al.’s approach to classifying errors. In this instance, we did not find that standing led to a more meticulous search than sitting, but that standing led to a sharper increase in errors across set size than sitting – a finding similar to that of Smith et al. – suggesting that, if anything, standing harmed performance as set size increased. That said, given that the effect size is quite small in comparison to the one reported by Smith et al. and that this effect is not observed when analyzing the percentage error data using a conventional approach to classifying errors, we believe it is most likely not meaningful and thus refrain from providing a speculative interpretation.

**Figure 5:**
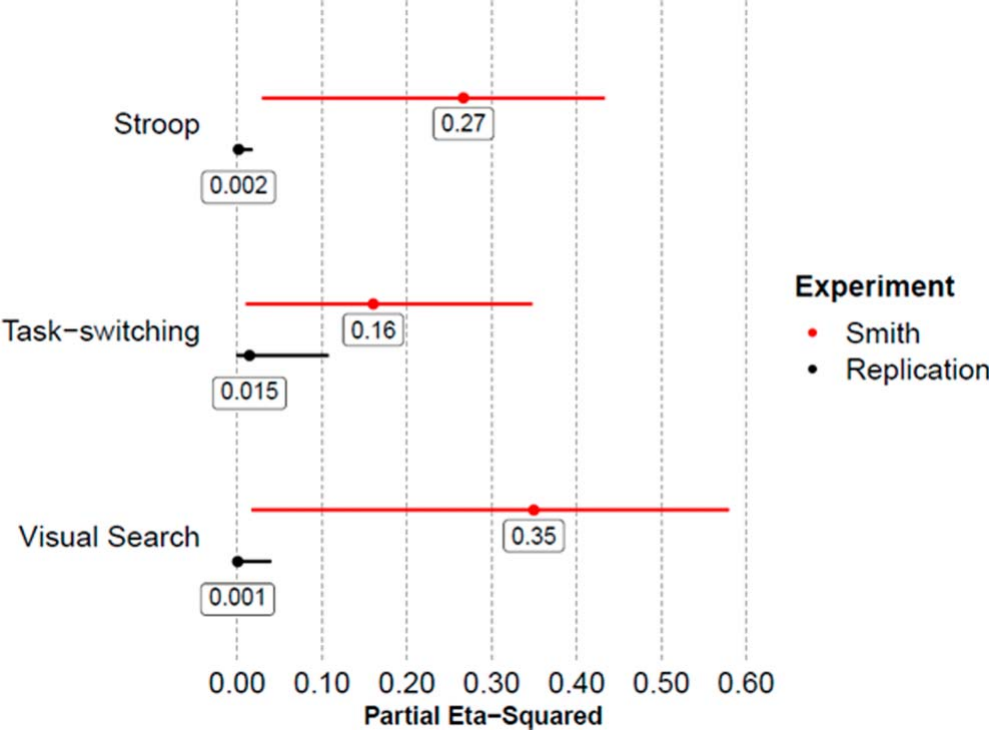
Forest plot illustrating the effect sizes of the postural interactions for the RT data reported in three Smith et al. (2019) experiments, as well as the postural effect sizes for three RT data obtained across the three replication experiments reported here. Error bars represent 95% confidence intervals.

When reviewing the effect sizes of the interactions from the response times and percentage error analyses with those from the three Smith et al. experiments (Figure 5), we noted two differences. First, the magnitude of the effect sizes observed across the three replications are a fraction (average of 3.28%) of Smith et al.’s initially reported effect sizes of interest. Second, the confidence intervals around the effect sizes here are much narrower than those around the effect sizes from the three Smith et al. experiments. Considering this in addition to reported failures to replicate by Caron et al. (2020) and Straub et al. (2022), it seems reasonable to presume that the effect of posture on cognitive performance in these tasks is not as robust as was initially observed in the original Smith et al. report.

The most likely explanation for the discrepancy across these studies might involve statistical power. Specifically, the difference in results might be attributable to the fact that the present studies had higher power (N_analyzed_ = 50 for Experiments 1 and 3, and N_analyzed_ = 51 for Experiment 2; a total of N_analyzed_ = 151) than those reported by Smith et al. (2019; with samples of N_analyzed_ = 14, 30, and 12, respectively; a total of N_analyzed_ = 56). Evidence has previously shown that studies with low power can produce unstable and often inflated effects (e.g., Button et al., 2013; Gelman & Carlin, 2014; Schönbrodt & Perugini, 2013). Our failure to replicate the authors’ original findings could therefore be due to inflated effects in the original work. It is worth noting, however, that in both our experiments and the Smith et al. experiments, there was sufficient statistical power to obtain the standard effects found in these common cognitive tasks (i.e., main effects of congruency in Experiment 1, switching and congruency in Experiment 2, and set size in Experiment 3; see https://osf.io/kwrjd).

Notably, although we attempted to replicate the experimental methods of Smith et al. as closely as possible, there will always be unavoidable differences across studies due to variations in time, laboratories, and participant pools. The very large difference in effect sizes across the postural interactions from this paper and those reported by Smith et al. (2019) make it unlikely, however, that simple variations in time, laboratories, and participant pools are the sole cause of these statistical discrepancies.

In all, the current research failed to provide support for Smith et al.’s (2019) theory that an individual’s attention improves when standing because of moderate increases in arousal or an association between this posture and specific mental states such as those involved in the fight-or-flight response. These findings also fail to support Rosenbaum et al.’s (2017/2018) theory that standing increases *load* leading to fewer resources being available to process the distractor words in the Stroop task. Admittedly, we only tested two postures across three cognitive control tasks, and we have not exhaustively examined the influence of posture in all available cognitive paradigms. Thus, interactions among the body and bodily states must still be investigated in the context of other cognition processes. Future research on posture, and more generally the body, bodily states, and cognition should include high statistical power, and perhaps, also seek to identify potential moderating factors for such effects.

## Footnotes

^1^
While we were unable to find information regarding whether Smith et al. (2019) included practice trials in their description of Experiment 3, we had participants complete one practice session of eight trials. This decision was made with the consideration that Rosenbaum et al. (2017/2018) had only included a single set of practice trials in their prior experiments.

## Notes

**Publication Ethics:** Informed consent was obtained from all participants included in the study. All procedures in studies involving human participants were performed in accordance with the ethical standards for the respective institutions. Ethics approval for Experiments 1 and 3 was provided by a Trent University Research Ethics Committee (ORE #22305 and #21948) and a University of Waterloo Research Ethics Committee (#31602). For Experiment 2, ethics approval was provided by a Texas A&M University-Commerce Research Institutional Review Board (#2218) and by the U.S. Army Research Laboratory (21‐126). A data sharing agreement between Trent University and the U.S. Army Research Laboratory was signed permitting the U.S. Army Research Laboratory to access nonidentifiable data from Experiments 1 and 3 prior to its publication on OSF (https://osf.io/kwrjd).

**Authorship:** Shulan Lu and Michael G. Reynolds share last authorship position. Conceptualization: Emilie E. Caron, Jonathan S. A. Carriere, Daniel Smilek, Shulan Lu, and Michael G. Reynolds; Data curation: Emilie E. Caron, Laura R. Marusich, Jonathan Z. Bakdash, Derek Harter, and Michael G. Reynolds; Formal analysis: Emilie E. Caron, Laura R. Marusich, Jonathan Z. Bakdash, Derek Harter, and Michael G. Reynolds; Funding acquisition: Emilie E. Caron, Jonathan S. A. Carriere, Daniel Smilek, Derek Harter, Shulan Lu, and Michael G. Reynolds; Investigation: Reynolds J. Ballotti, Andrew M. Tague, Derek Harter, Shulan Lu, and Michael G. Reynolds; Methodology: Emilie E. Caron, Laura R. Marusich, Jonathan Z. Bakdash, Reynolds J. Ballotti, Andrew M. Tague, Jonathan S. A. Carriere, Daniel Smilek, Derek Harter, Shulan Lu, and Michael G. Reynolds; Project administration: Emilie E. Caron, Laura R. Marusich, Jonathan Z. Bakdash, Shulan Lu, and Michael G. Reynolds; Resources: Derek Harter, Shulan Lu, and Michael G. Reynolds; Software: Laura R. Marusich, Jonathan Z. Bakdash, Derek Harter, and Michael G. Reynolds; Supervision: Emilie E. Caron, Reynolds J. Ballotti, Jonathan S. A. Carriere, Daniel Smilek, Shulan Lu, and Michael G. Reynolds; Validation: Emilie E. Caron, Laura R. Marusich, Jonathan Z. Bakdash, Derek Harter, Shulan Lu, and Michael G. Reynolds; Visualization: Emilie E. Caron, Laura R. Marusich, Jonathan Z. Bakdash, Derek Harter, and Michael G. Reynolds; Writing – original draft: Emilie E. Caron, Laura R. Marusich, Jonathan Z. Bakdash, and Derek Harter; Writing – review & editing: Emilie E. Caron, Laura R. Marusich, Jonathan Z. Bakdash, Daniel Smilek, Shulan Lu, and Michael G. Reynolds.

**Open Data:** To the best of my ability and knowledge, I have provided all original materials and clear references to all other materials via a stable online repository. *Open Data:* The data and code to reproduce all reported results are available at https://osf.io/kwrjd (Caron et al., 2022) and on Code Ocean: https://doi.org/10.24433/CO.1763342.v2. *Open Materials:* Available at https://osf.io/kwrjd under Code to run experiments component (Caron et al., 2022). The online supplementary materials are available at https://osf.io/kwrjd, while the preprint is available at https://osf.io/dfgwy. A full list of all R packages used is provided in the online supplemental materials. *Pre-registration and Analysis Plan:* My manuscript contains at least one experiment with a completely executed pre-registration or explicit notes on any deviation from the pre-registration. The experimental designs and analysis plans were pre-registered at https://osf.io/fzscv.

## Appendix

Here, we report the results from analyzing all three experiments’ PE data using a conventional approach; that is, trials that exceeded the time-out threshold were dropped from the analysis, rather than being included as errors.

### Experiment 1: Stroop

The results from the conventional PE analyses for Experiment 1 demonstrate a significant main effect of congruency, *F*(2, 98) = 8.81, *MSE* = 9.64, *p* < .001, η_*p*_^2^ = .152, but no significant main effect of posture, *F*(1, 49) = 0.03, *MSE* = 14.82, *p* = .868, η_*p*_^2^ < .001, or interaction of posture by congruency, *F*(2, 98) = 1.53, *MSE* = 5.87, *p* = .221, η_*p*_^2^ = .030, BF = 6.93, *p*BIC(H0|D) = 0.14. The results from the reported Bayes factor (BF) reveal that there was nearly 7 times more evidence supporting the null than the alternative hypothesis.

### Experiment 2: Task-Switching

In Experiment 2, conventional PE analyses demonstrate significant main effects of switching, *F*(1, 50) = 99.91, *MSE* = 21.86, *p* < .001, η_*p*_^2^ = .666, and congruency, *F*(1, 50) = 108.95, *MSE* = 76.69, *p* < .001, η_*p*_^2^ = .685; but no significant main effect of posture, *F*(1, 50) = 0.65, *MSE* = 77.65, *p* = .42, η_*p*_^2^ = .01. The results further show a significant congruency by switching interaction consistent with Smith et al. (2019), *F*(1, 50) = 63.78, *MSE* = 13.78, *p* < .001, η_*p*_^2^ = .561, but no significant switching by posture interaction, *F*(1, 50) = 1.40, *MSE* = 18.61, *p* = .24, η_*p*_^2^ = .027, BF = 5.83, *p*BIC(H0|D) = 0.17. The BF reported here suggests that there is nearly 6 times more evidence favoring the null hypothesis than the alternative. No other significant interactions were observed.

### Experiment 3: Visual Search

Conventional PE data analyses for Experiment 3 demonstrate no significant main effect of set size, *F*(1, 49) = 2.31, *MSE* = 3.63, *p* = .135, η_*p*_^2^ = .045, and also no significant effect of posture, *F*(1, 49) = 3.31, *MSE* = 2.43, *p* = .075, η_*p*_^2^ = .063. When employing the conventional approach to classifying errors, no significant interaction of posture by set size was observed, *F*(1, 49) = .62, *MSE* = 2.79, *p* = .434, η_*p*_^2^ = .013, BF = 3.95, *p*BIC(H0|D) = 0.25. The BF reported here suggests that there was nearly 4 times more evidence favoring the null hypothesis than the alternative.
